# Respiratory Protection Perceptions among Malian Health Workers: Insights from the Health Belief Model

**DOI:** 10.3390/ijerph19053028

**Published:** 2022-03-04

**Authors:** Stella E. Hines, Joanna Gaitens, Nora M. Mueller, Diego Molina Ochoa, Eseosa Fernandes, Melissa A. McDiarmid

**Affiliations:** 1 Division of Occupation and Environmental Medicine, University of Maryland School of Medicine, Baltimore, MD 21201, USA; jgaitens@som.umaryland.edu (J.G.); mmcdiarm@som.umaryland.edu (M.A.M.); 2 Department of Social and Behavioral Sciences, Harvard T.H. Chan School of Public Health, Boston, MD 02115, USA; mueller.nm@gmail.com; 3 University of Maryland School of Medicine, Baltimore, MD 21201, USA; diego.molinaochoa@som.umaryland.edu; 4 Department of Preventative Medicine, University of Maryland School of Medicine, Baltimore, MD 21201, USA; eseosa.fernandes@som.umaryland.edu

**Keywords:** health behavior, quality of care, community and public health, health promotion, interprofessional health care, health care work environment, immunization and infection prevention, medical technology, well-being

## Abstract

Reusable respiratory protective devices called elastomeric respirators have demonstrated their effectiveness and acceptability in well-resourced healthcare settings. Using standard qualitative research methods, we explored the feasibility of elastomeric respirator use in low- and middle-income countries (LMIC). We conducted interviews and focus groups with a convenience sample of health workers at one clinical center in Mali. Participants were users of elastomeric and/or traditional N95 respirators, their supervisors, and program leaders. Interview transcripts of participants were analyzed using a priori constructs from the Health Belief Model (HBM) and a previous study about healthcare respirator use. In addition to HBM constructs, the team identified two additional constructs impacting uptake of respirator use (system-level factors and cultural factors). Together, these framed the perceptions of Malian health workers and highlighted both facilitators of and barriers to respirator use uptake. As needs for respiratory protection from airborne infectious hazards become more commonly recognized, elastomeric respirators may be a sustainable and economic solution for health worker protection in LMIC.

## 1. Introduction

With each global infectious disease outbreak, shortages in the supply of single-use disposable N95 filtering facepiece respirators (N95 FFRs) become evident. N95 FFRs, which filter out at least 95% of 0.3 µ sized airborne particles, are used in daily clinical work by healthcare providers and staff and are generally considered to be the first line of defense against airborne infectious disease. During the 2003 SARS, 2009 H1N1 influenza, and 2015 Ebola events, shortages in these respirators occurred both in well-resourced nations and in low- and middle-income countries (LMICs) [1,2,3,4]. During the COVID-19 pandemic, global respiratory protection device (RPD) shortages occurred again and resulted in the re-use of single-use N95 FFRs or the use of non-medical grade masks and face-shields by medical staff, potentially leaving health workers exposed to infectious pathogens and vulnerable to deadly illness [5]. This vulnerability highlights the need for innovative, sustainable, and cost-effective solutions for assuring ready access to respiratory protective equipment during shortages.

**Figure 1:**
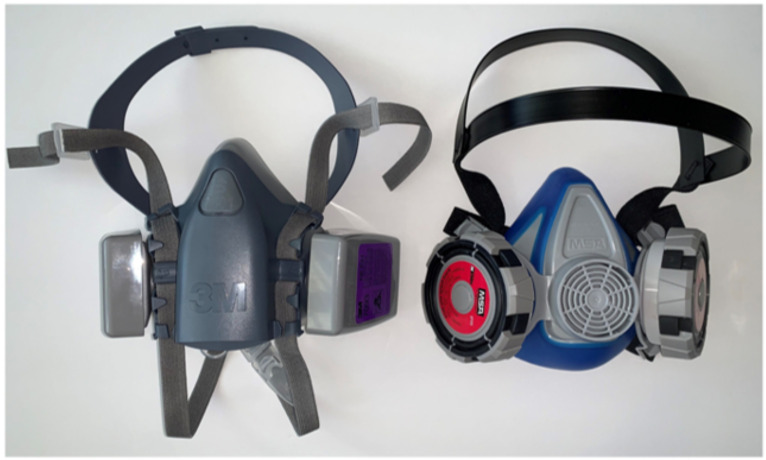
Examples of elastomeric respirators.

One solution for averting the lack of access to N95 FFRs during crises could be a reusable device. One type of reusable device is called an elastomeric respirator (Figure 1). Elastomeric respirators are tight-fitting face masks made of soft, conformable, synthetic or rubber material that contain ports to attach to protective filters [6]. Elastomeric respirators provide the same or higher level of protection as do single use N95 FFRs but can be cleaned and reused. Because they are reusable, healthcare facilities and workers do not have to rely on stockpiling of N95 FFRs or an unstable supply chain. Elastomeric respirators can be used and cleaned repeatedly, allowing for multiple uses [7]. Furthermore, prior research has shown that elastomeric respirators are acceptable to health workers in a U.S. hospital system [8].

Although they found limited use in well-resourced healthcare systems prior to the COVID-19 pandemic, elastomeric respirators have not previously been used in LMICs, with one known exception—The Center for Vaccine Development-Mali (CVD-Mali). Located in Bamako, Mali, this center partners with international colleagues to support vaccine and public health-related research trials and patient care [9]. In 2017, the center embarked upon an international childhood mortality study that required health workers to retrieve the bodies of deceased children from the community and perform limited postmortem testing to assess for causes of death [10]. These tasks could potentially expose staff to infectious aerosols. To protect health workers, an occupational health faculty member from a US-based university partner traveled to the center and trained and fit-tested staff to wear elastomeric respirators. Some of those trained, who already possessed safety or infection control backgrounds, then became respiratory protection program “champions” and tested and trained other staff. This 2017 elastomeric respirator training effort included completion and review of a medical clearance questionnaire, qualitative fit-testing, and verbal instructions and demonstration on how to don, doff, and clean the respirator. More than 35 Malian staff (including pathologists, laboratory workers, and clinicians) participated in the elastomeric respirator-based respiratory protection program (RPP).

Following the 2017 training session, additional staff who would be interacting with community members as part of the study during the consenting and retrieval process for the deceased were designated by the local Malian team to wear N95 FFRs instead of elastomeric respirators. These community-facing staff members were not assigned or trained to use elastomeric respirators. The appearance of N95 FFRs, which look similar to traditional surgical masks in popular use, were thought to be more acceptable to the public than elastomeric respirators and to be more appropriate for interacting with family and community members. Since the 2017 launch, the RPP had not been formally evaluated to understand how well it was working.

One method for assessing the user perspective of a health behavior, such as use of a respirator, is the Health Belief Model (HBM). First described in 1952, the model includes domains of perceived susceptibility, severity, benefits and barriers, perceived threat, cues to action, self-efficacy, and demographic factors to understand whether a protective health action is taken [11]. The HBM has found some applications in understanding attitudes toward protection from respiratory infections. A 2006 study applied the HBM to understand family physician perceptions on PPE use following the SARS outbreak in Singapore, finding that PPE use was driven by perceived threat [12]. A 2014 narrative review of facemask use for preventing respiratory infections found that perceived susceptibility and severity were key determinants of compliance with the use of facemasks [13]. A 2019 study also found that although U.S. health workers recognize the importance of respiratory protection, this knowledge gets undermined by the realities of clinical practice, prior experience, and perception of risk, leading to lapses in consistent practice [14]. While previously used to understand these respiratory protection beliefs in higher income countries, the HBM likely also can shape understanding of health worker opinions in LMICs.

Health worker opinions may be evaluated using qualitative research methods, such as focus group interviews. Focus group interviews help to understand how people feel or think about an issue [15]. When applied to the issue of respiratory protection, organizations and researchers may apply this information to design better respiratory protection plans or survey instruments to use in future quantitative studies using statistical analysis. Thus, qualitative research methods play a key role in applied research about human behavior.

For this qualitative study, we conducted a framework analysis using constructs of the HBM. We analyzed transcripts of focus groups that collected data on the perceptions of health workers using elastomeric and N95 filtering facepiece respirators. A foundational understanding of the barriers and facilitators to elastomeric respirator use among health workers in this setting may facilitate the design, implementation, and optimization of RPPs not only there but also in other LMICs, enhancing the protection of health workers during times of uncertain PPE supply.

## 2. Methods

### 2.1. Focus Group Interviews

The study team developed focus group interview scripts related to respiratory protection based on previous interviews of hospital respiratory protection leaders [16]. These scripts were refined following discussions among study team members to incorporate the current understanding of the structure of the center’s respirator program. The purpose of the focus group interviews was to validate the structure and operation of the center’s RPP, as well as to elicit perceptions of respirator use among health workers. The study team crafted separate interview scripts for three different groups: RPP champions, supervisors, and respirator users. The study team then sent the final versions of the interview scripts to the center’s respiratory protection champions who translated them into French.

The study team requested interviews with both worker populations enrolled in the RPP—those who used elastomeric respirator and N95 FFR users—as well as their supervisors and the champions of the RPP. RPP champions arranged times for the in-person focus group interviews and assured availability of the respirator users and supervisors. Respirator users and supervisors worked in the departments of pathology, microbiology, and immunology or other program-specific areas such as family communication, body preparation for burial, and environmental hygiene. Over a series of three days in February 2020, five focus groups met. Each focus group was led by the same study team member, with two additional study team members participating in support roles and taking notes. In each group, the focus group leader explained the purpose of the session to participants and asked for permission to record the session and to transcribe and subsequently review notes. All participants agreed to being recorded. The first mini-focus group included two RPP leaders or champions, and the second mini-focus group included three supervisors. The three additional focus groups consisted of mixed populations of elastomeric and N95 respirator users. These three focus groups were structured to include approximately 10 respirator users per group, as per ideal focus group size recommendations [15]. The groups varied in size between nine and eleven participants each. Supervisors or managers of the respirator users were not present during the respirator user interviews. A French-speaking translator assisted when needed. Each focus group required 1–1.5 h. The English language portions of the recordings were transcribed verbatim. The study team’s university institutional review board determined that the intended project was not considered human subjects research, but rather quality improvement. Therefore, additional approval was not required. The respiratory protection program evaluation was funded through a contract with the U.S. Centers for Disease Control and Prevention (CDC) National Institute for Occupational Safety and Health (NIOSH).

### 2.2. Analysis

Using qualitative methods to assess focus group responses, five study team members read all focus group transcripts to review the data [17]. Using the interview scripts to provide the initial structure, we created an a priori codebook of topics for classifying interview transcript statements. We further included constructs from the HBM to capture individual behaviors and motivations for respirator use. Findings were framed under six components of the HBM: perceived susceptibility, perceived severity, perceived benefits, barriers to action, cues to action, and self-efficacy. In this context, the action in question was respirator use. Therefore, evaluating barriers or cues to action would include evaluating barriers or cues to using respirators. However, as noted in prior studies, the HBM does not consider the impact of complex social systems on individual behavior and may be of limited use when analyzing the behavior of health workers [14]. Therefore, we included additional domains of system-level and cultural factors that promoted or prevented adherence to respiratory protection guidelines during our analysis.

Using NVivo 12 qualitative analysis software (QSR International Pty Ltd., Version 12, 2018), three study team members then approached the data formally using both an inductive and deductive approach, capturing a priori themes but also those that presented themselves during coding [17,18]. The five-person team reviewed all coding and iteratively collapsed codes into larger groups. Once completed, they independently reviewed coding and crafted memos, or synopsis notes, summarizing the findings. The study team then convened to discuss each member’s memo, asked clarifying questions, and highlighted any newly evident themes. This occurred until discussions reached consensus. Study team members then revised their memos and submitted them to the qualitative lead, who crafted a summary memo. This process recurred until all thematic categories were reviewed. Finally, the group reviewed all summary memos to assure that each of the key themes had been summarized in accordance with the group discussions. Overlap between key themes was common; when this occurred, the team classified text in the theme that it best represented. The study team group discussions occurred during a series of six meetings, typically lasting 1.5 h each.

## 3. Results

### 3.1. Sample Overview

A total of 35 individuals participated in five focus groups (Table 1), representing all individuals participating in the RPP at the time. Focus groups of health workers included 30 respirator users involved with the childhood mortality study, (laboratory workers, physicians, body washers, hygienists, and social and behavioral scientists); the supervisor group included directors of three laboratories, and the RPP Champions included two respiratory protection leaders.

### 3.2. Themes

#### 3.2.1. Cultural Factors Impacting Respirator Use

Supervisors stated that all staff were aware of and adhered to the expected respiratory protection practices. Although elastomeric respirators are used by certain workers while physically working at the center, they are not worn when staff are working in the community performing tasks associated with the deceased collection protocol. In the community, RPP champions stated that it is expected that N95 FFRs are worn. This includes during consent and when transporting the deceased to the ambulance, and again upon arrival at the study site for washing the body and sampling and processing specimens. Social and behavioral scientists, who make first contact with the family of the deceased child, wear simple dust masks that they purchase with their own money when they ride a motorbike to a forensics scene. Even though dust masks seem to be generally accepted in the community, there was a suggestion that other types of masks, whether N95 FFR or elastomeric respirator, may raise concern, because community members may feel that “*there is something bad*”. Respirator program champions also expressed concern that respirator use by the team—regardless of respirator type—might be seen as disrespectful and might convey differential equity in availability of protection, saying, “*If you wear a mask, you are more protected than them.*” Several participants noted that wearing a mask creates a cultural barrier between the worker and the community member. One suggested wearing a mask indicated “*you don’t have enough respect for that person*”.

#### 3.2.2. System-Level Factors Impacting RP Use

CVD Mali and its U.S.-based University partner have an established relationship that supports the center’s RPP. RPP champions stated that because of this relationship, no financial strains limit their ability to provide safety equipment. However, participants from the worker group reported reusing N95 FFRs that were meant to be disposable, attributing that behavior to being “good citizens” and saving the program money.

The interviews showed several system-level communication and training issues. For example, when discussing the process of disinfecting the elastomeric respirators, RPP champions understood that the respirators were disassembled after each use by hygienists and disinfected by submersion in a bleach solution. Instead, hygienists and laboratory workers reported disinfecting the respirators with disposable tissues and 70% alcohol and not always removing the filter cartridges. This discrepancy between how the leaders understood the RPP to be functioning and the process in practice reflects a systems issue in quality assurance. As another example, respirator users are supposed to ask questions when clarification is needed, but there appears to be a hierarchy to follow, and it is unclear who is responsible for answering questions. Training is reported to be sporadic, and it is unclear who is responsible for training. Although a few new workers received informal training by other co-workers, no formal fit-testing or training had occurred since the 2017 program launch. Additionally, none of the N95 FFR users ever received formal fit-testing.

### 3.3. Perceived Susceptibility

The 2015 Ebola epidemic played a notable role in perceived susceptibility of these workers toward infection. Staff recalled the illness and death of a physician colleague during the prior Ebola crisis. This recognition was a cue that if they got sick, they could infect their family members (Box 1). This awareness of the ability to affect others outside of their work, and in their homes, mosques, and communities was cited often.

Box 1 **Perspective on perceived susceptibility for focus group participant.**

*“But with Ebola everybody understands that if you don’t wear the PPE correctly, you are at risk and maybe your family is at risk, maybe your colleague is at risk, maybe your colleagues at the mosque are at a risk, maybe everybody around you, even in the market, in the car, in the bus are at a risk. We keep that in our minds, and even greeting someone with your hand is taking the risk. Today, myself even at the mosque after prayer I never took the hand of somebody because of our training by Ebola that this is the risk.”*

RPP Champion

### 3.4. Perceived Severity

In addition to their own perceived susceptibility, perceived severity of impact from not wearing a respirator was reflected by some. There was recognition, for example, that death was a potential consequence from not wearing a respirator during care of patients with Ebola. Notably, however, there was a discrepancy between the risk the staff observed from a more common infection, tuberculosis, compared with how they thought the risk was perceived by the public. As one person noted, “*they [the public] are not concerned about it, but among health worker—they know how it is and what it is, how it is to be sick from that and the difficulty*”.

### 3.5. Perceived Benefits

Interviewees consistently noted benefits to use of respirators, including protecting not just oneself, but family, friends, and community members. Elastomeric respirator users stated that they know the mask protects because they can perform a fit check and assure that the air is being pulled through the filters. They reported that even when handling a decomposed body, the elastomeric respirator users do not detect any odor. Several staff, assigned to wear N95 FFRs, expressed that they wished they could wear the elastomeric respirator instead of the N95 FFRs due to this perceived benefit. Elastomeric users referred to the masks as providing “*maximum protection*”. N95 FFR users report easy breathing, simplicity of use, and not scaring community members as benefits. Notably, both RPP champions and supervisors associated respirator use with protection of laboratory samples from source contamination and assurance of the integrity of the specimens, although the purpose of respiratory protection is staff, not specimen, protection.

### 3.6. Barriers to Action

For elastomeric respirator users, barriers to action (such as using respirators) include reported difficulty breathing, discomfort when the respirator is too tight, and difficulty in donning. Another perceived barrier for elastomeric respirator use was understanding of whether respirators were to be assigned to specific individuals or available to any of the trained, fit-tested users as a part of a generic cache. Other comments included, “*it changes your voice*”, “*it’s not part of the behavior*”, and it creates “*difficulty to recognize even your face*”. For N95 FFR users, barriers to using respirators included respirators “*smelling*” and being able to smell the dead bodies when they are being washed. At least one N95 FFR user felt the masks did not protect enough.

### 3.7. Cues to Action

One of the specific cues for respirator use described by the interviewees was the death of a colleague physician in 2015 from Ebola. This was highlighted as a reminder of vulnerability and severity of illness, but also as a cue to wear the respirator to prevent illness. Otherwise, participants did not describe visible cues to wearing respirators, such as signs or posted guidelines. The site was, however, crafting a biosecurity plan at the time of the interviews. Respirator users asked questions during the interviews about respirator use, clearly showing an interest in learning more, along with knowledge deficits.

### 3.8. Self-Efficacy

By having PPE and respiratory protection available, staff recognized that they had the power to protect themselves and others (Box 2). At no point during the interviews did staff reflect a concern of being unable to carry out their jobs due to lack of protection.

Box 2 **Perception on self-efficacy expressed by focus group participant.**

*“It will allow us to live our life during longtime and this is why I think it’s great to use the respirator to be protective and protect your neighbor, protect your colleague, protect your family and even protect the community.”*

RPP Champion

**Figure 2:**
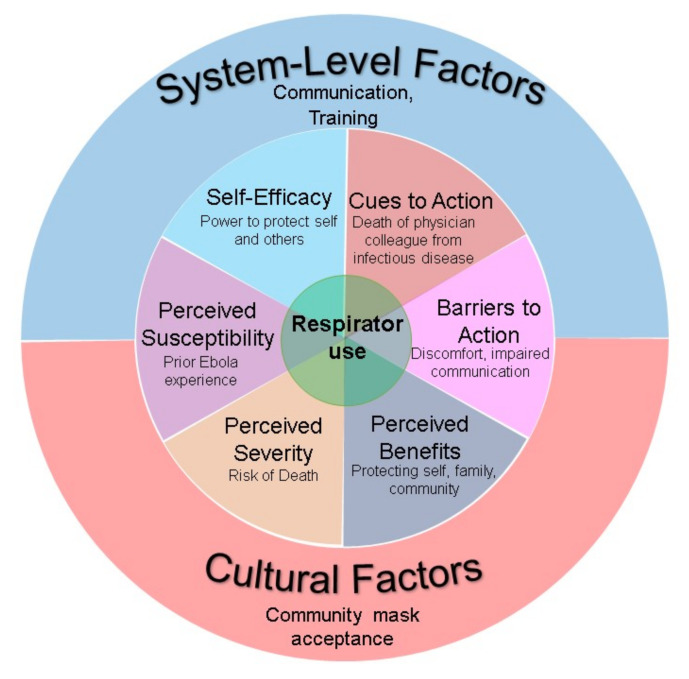
Framework including the Health Belief Model applied to health worker perspectives about respirator use.

Figure 2 summarizes how the Health Belief Model and system-level and cultural factors framed the understanding of Malian health worker perspectives on respirator use.

## 4. Discussion

Using qualitative research methods, we applied the HBM, supplemented with influential cultural and social factor components as a framework for understanding the opinions and experiences of Malian health workers related to respirator use. Most consistently, the HBM constructs of perceived susceptibility and severity of possible illness, and self-efficacy in assuring their own protection, illustrated interviewee opinions around respirator use. In general, respirator use was acceptable to these health workers, as it was perceived to provide protection for them. By preventing their own illness, they could also protect their families and community. Although referenced as harder to breathe through and possibly not acceptable to be worn in a community setting, many interviewees perceived that the elastomeric respirators provided greater protection than the N95 FFRs. This perception also facilitated uptake of this novel form of respiratory protection.

Our findings share similarities to other reports using the HBM to assess attitudes about respiratory protection. In prior studies, perceived susceptibility and severity of disease risk significantly influenced workers’ likelihood of wearing face masks and respirators to prevent acute respiratory infections, including SARS [12,13,14]. These domains outweighed perceived barriers, such as discomfort or cost [12]. Similarly, U.S.-based health workers interviewed about their preferences for elastomeric respirators versus N95 FFRs in a 2017 qualitative analysis perceived that benefits such as greater protection outweighed perceived barriers such as discomfort [16]. Overall, these findings support the belief that health workers will adopt respiratory protection—including use of elastomeric respirators—in line with cultural influences and system-level constructs suggested from the HBM.

The findings from these interviews identified successes, such as sense of protection, and challenges, such as cleaning and disinfection logistics, similar to those seen in well-resourced RPPs, including elastomeric respirator-based programs. Overall, user acceptance in this small, West African health worker population is predominantly positive. Given this first success, elastomeric respirator use could be a sustainable respiratory protection option in other low resource settings.

Our study has several limitations and strengths. First, as is usual with qualitative studies, our findings are particular to this setting and group and may not be generalizable. However, the consistency between these health workers’ opinions with those observed in other published studies from respirator users provides reassurance that these findings have broader applications. Second, we were limited in applying the “cues to action” framework to our data, as no scripted interview questions specifically addressed this topic. Even so, interviewees provided responses to other questions that addressed this domain of the HBM. Third, this analysis occurred at a research center with international support and not at a general health facility in Mali. Results may not be generalizable to more typical Malian or LMIC healthcare settings. Similarly, as focus group participants worked in a setting that required use of masks in the community, the cultural barriers observed in this study likely differ from those in hospital settings, where masks may be more commonly used and rapport with patients and families would permit better understanding of their use. Additional study including a quantitative survey assessment would provide insight on user acceptance, respirator use patterns, and RPP logistics from this setting, with potential applications to other LMIC healthcare environments.

## 5. Conclusions

In summary, the utility of domains of the HBM for assessing respirator user perceptions in well-resourced settings also aptly frame the perceptions of health workers in an LMIC regarding their use of respiratory protection. These health beliefs and perceptions include those that both facilitate uptake and may act as barriers toward respirator use. As airborne hazard threats to health workers are expected to continue, reusable elastomeric respirators appear to provide a practical and sustainable solution to respiratory protection needs in LMIC settings.

## Figures and Tables

**Table 1:** Focus group participants.

Focus Groups					
Group Type	RPP Champions	Supervisors	Respirator Users Group 1	Respirator Users Group 2	Respirator Users Group 3
N& gender	N = 2; 2 men	N = 3; 1 woman, 2 men	N = 9; 2 women, 7 men	N = 10; 3 women, 7 men	N = 11; 2 women, 9 men

## Data Availability

The data presented in this study are available on request from the corresponding author.
